# In Vivo Analysis of the Regeneration Capacity and Immune Response to Xenogeneic and Synthetic Bone Substitute Materials

**DOI:** 10.3390/ijms231810636

**Published:** 2022-09-13

**Authors:** James Bielenstein, Milena Radenković, Stevo Najman, Luo Liu, Yanru Ren, Baoyi Cai, Florian Beuer, Denis Rimashevskiy, Reinhard Schnettler, Said Alkildani, Ole Jung, Franziska Schmidt, Mike Barbeck

**Affiliations:** 1 Clinic and Policlinic for Dermatology and Venereology, University Medical Center Rostock, 18057 Rostock, Germany; 2 Department for Cell and Tissue Engineering, Scientific Research Center for Biomedicine, Faculty of Medicine, University of Niš, 18000 Niš, Serbia; 3 Department of Biology and Human Genetics, Faculty of Medicine, University of Niš, 18000 Niš, Serbia; 4 Beijing Advanced Innovation Center for Soft Matter Science and Engineering, College of Life Science and Technology, Beijing University of Chemical Technology, Beijing 100013, China; 5 Department of Prosthodontics, Geriatric Dentistry and Craniomandibular Disorders, Charité-Universitätsmedizin Berlin, Corporate Member of Freie Universität Berlin, Humboldt-Universität zu Berlin, and Berlin Institute of Health, 14197 Berlin, Germany; 6 Department of Traumatology and Orthopedics, Peoples’ Friendship University of Russia, 117198 Moscow, Russia; 7 University Medical Centre, Justus Liebig University of Giessen, 35390 Giessen, Germany; 8 BerlinAnalytix GmbH, 12109 Berlin, Germany

**Keywords:** xenograft, synthetic graft, bone grafts, immune response, macrophage polarization, DIN ISO 10993-6, immunohistochemical staining, bone regeneration

## Abstract

Although various studies have investigated differences in the tissue reaction pattern to synthetic and xenogeneic bone substitute materials (BSMs), a lack of knowledge exists regarding the classification of both materials based on the DIN ISO 10993-6 scoring system, as well as the histomorphometrical measurement of macrophage subtypes within their implantation beds. Thus, the present study was conducted to analyze in vivo responses to both xenogeneic and synthetic bone substitute granules. A standardized calvaria implantation model in Wistar rats, in combination with established scoring, histological, histopathological, and histomorphometrical methods, was conducted to analyze the influence of both biomaterials on bone regeneration and the immune response. The results showed that the application of the synthetic BSM maxresorb^®^ induced a higher pro-inflammatory tissue response, while the xenogeneic BSM cerabone^®^ induced a higher anti-inflammatory reaction. Additionally, comparable bone regeneration amounts were found in both study groups. Histopathological scoring revealed that the synthetic BSM exhibited non-irritant scores at all timepoints using the xenogeneic BSM as control. Overall, the results demonstrated the biocompatibility of synthetic BSM maxresorb^®^ and support the conclusion that this material class is a suitable alternative to natural BSM, such as the analyzed xenogeneic material cerabone^®^, for a broad range of indications.

## 1. Introduction

A broad spectrum of bone substitute materials (BSMs) has been developed and evaluated in recent decades. Two main BSM classes have been established for use in daily clinical practice. These include so-called “natural” materials that originate from both allogeneic and xenogeneic bone tissue, as well as synthetic materials, which form part of the clinical portfolio. Xenogeneic materials have been applied for different indications. Synthetic biphasic BSMs have also been shown to provide comparable regenerative properties for the same indications, such as sinus augmentation procedures [1,2].

Interestingly, both BSM types have been observed to undergo two different biodegradation behaviors. Two different degradation mechanisms have been identified for calcium phosphate-based BSMs: (i) cellular bioresorption and (ii) dissolution within the surrounding body fluid [3,4]. “Natural” BSMs, such as xenogeneic materials that are mainly based on hydroxyapatite (HA), tend to have a lower biodegradation pattern and an associated higher volume stability, while materials based on beta-tricalcium phosphate (β-TCP), also combined with HA in the form of biphasic BSMs, show faster degradation behavior [5]. In this context, the cellular degradation pathway has been discussed and analyzed in several studies [6,7,8]. Both material types induce a tissue reaction cascade mainly involving macrophages and multinucleated giant cells (MNGCs), jointly comprising phagocytes, that phagocytose parts of implanted materials [9,10]. The main difference between the two BSM classes is that xenogeneic materials have been shown predominantly to induce phagocytes in the early post-implantation phase, with their occurrence decreasing after some weeks as the xenogeneic BSM materials become embedded within the bone matrix, without leaving major surface areas for phagocytic activities [9,10]. In contrast synthetic materials have been found to induce a much higher tissue response which persists over longer time periods and includes higher phagocyte numbers [9,10]. Thus, materials of this class undergo greater cellular degradation, and demonstrate higher solubility behavior based on their phase composition [9,10].

Apart from their phagocytosing activity, phagocytes have been shown to be involved in molecular cascades towards biomaterials via the expression of a broad variety of pro- and anti-inflammatory molecules whose overall alignment appears to determine the direction of the material-associated regeneration process [8,11,12]. Thus, the sum of the physicochemical characteristics of a biomaterial is influenced by the alignment of the cellular expression pattern and behavior [7,13,14]. In this context, biomaterials that generate a pronounced pro-inflammatory cell response are classified as “bioincompatible”, while materials that contribute to an overall anti-inflammatory cell cascade phenotype help to trigger bone tissue regeneration [11,12].

Although previous studies have examined differences in the tissue reaction pattern of both material classes, a lack of knowledge exists about the classification of both materials based on the DIN ISO 10993-6 scoring system, as well as the histomorphometrical measurement of macrophage subtypes within their implantation beds [3,4,6,9,10,15].

Thus, the present study was conducted to analyze in vivo responses to both xenogeneic and synthetic biphasic bone substitute granules. A standardized calvaria implantation model in Wistar rats, in combination with established histological, histopathological, and histomorphometrical methods, was conducted to analyze the influence of both biomaterials on bone regeneration and the immune response [16,17].

## 2. Results

### 2.1. Histopathological Analyses

**Figure 1:**
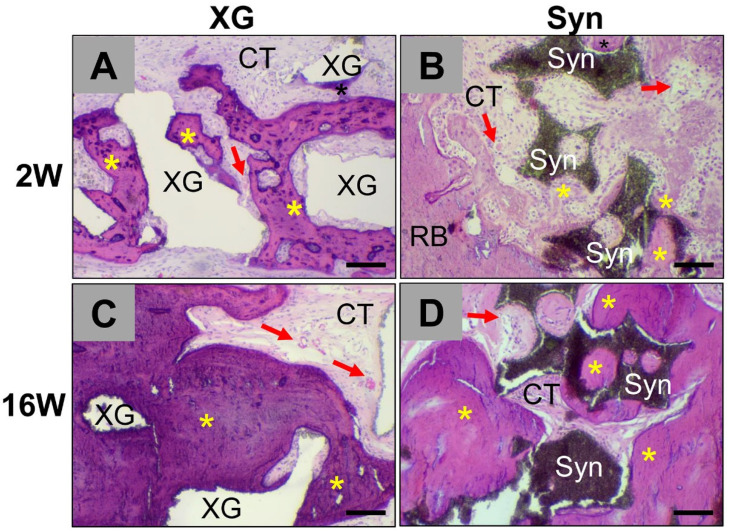
Overview of the implantation beds of the BSMs including (**A**,**C**) the xenogeneic BSM cerabone^®^ (XG), (**B**,**D**) the synthetic BSM maxresorb^®^ (Syn) at 2 weeks (**A**,**B**) and 16 weeks (**C**,**D**) post implantation. Red arrows = blood vessels, asterisks = new bone growth, CT = connective tissue. (H&E staining, 10× magnification, scale bars = 100 µm).

Histopathological analysis showed that both the xenogeneic (XG) and the synthetic substitute materials (maxresorb^®^ and cerabone^®^) were observable within defect regions at two and sixteen weeks post implantation (Figure 1).

**Figure 2:**
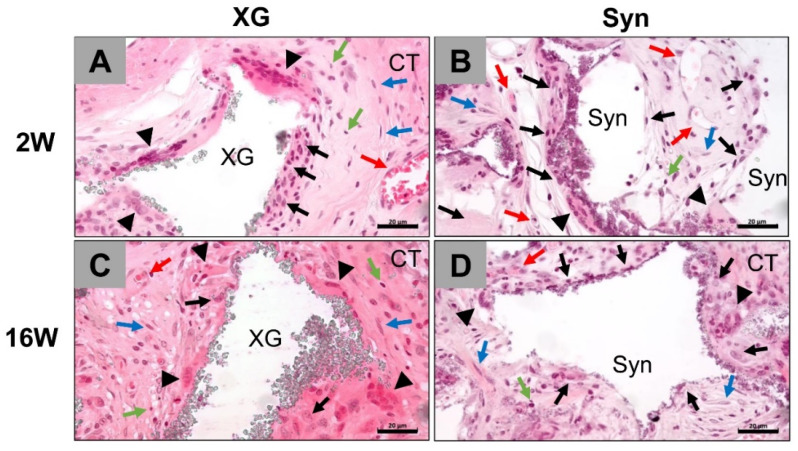
Tissue reactions to the BSMs include (**A**,**C**) the xenogeneic BSM cerabone^®^ (XG) and (**B**,**D**) the synthetic BSM maxresorb^®^ (Syn) at 2 weeks (**A**,**B**) and 16 weeks (**C**,**D**) post implantation. Red arrows = blood vessels, black arrows = macrophages, black arrowheads = multinucleated giant cells, blue arrows = fibroblasts, green arrows = lymphocytes, CT = connective tissue (H&E staining, 40× magnifications, scale bars = 20 µm).

At two weeks post implantation, slight bone matrix regeneration was observed within all implantation beds to a comparable extent outwards from the defect borders (Figure 1). A comparable moderate infiltrate of inflammatory cells, i.e., a granulation tissue, mainly comprising macrophages and lower numbers of polymorphonuclear cells, lymphocytes, and fibroblasts, in concert with a moderate vascularization pattern, was observable within the implantation beds of both BSMs (Figure 1 and Figure 2).

At the material surfaces of the xenogeneic and the synthetic BSMs macrophages were mainly detected as well as single multinucleated giant cells in comparable amounts in both study groups (Figure 1 and Figure 2). In this period, the presence of plasma cells or any scarring, necrosis, or fatty infiltrates was not observed in any study group.

At 16 weeks after implantation, a higher degree of bone regeneration was found in both study groups without visible differences (Figure 1). Increased osteoconductive bone growth was seen involving the surfaces of both the synthetic and xenogeneic BSMs (Figure 1 and Figure 2).

Tissue reactions involving mainly macrophages and lower numbers of polymorphonuclear cells, lymphocytes, and fibroblasts were observable in both study groups (Figure 1 and Figure 2). Both mono- and multinucleated phagocytes were predominantly found at the material surfaces of the synthetic bone granules, while slightly lower phagocyte numbers were observed on the surfaces of the xenogeneic bone granules (Figure 1 and Figure 2). Additionally, moderate implantation bed vascularization was observed in both groups to a comparable extent, while no presence of plasma cells or any scarring, necrosis, or fatty infiltrates was observed in any study group (Figure 1 and Figure 2).

**Figure 3:**
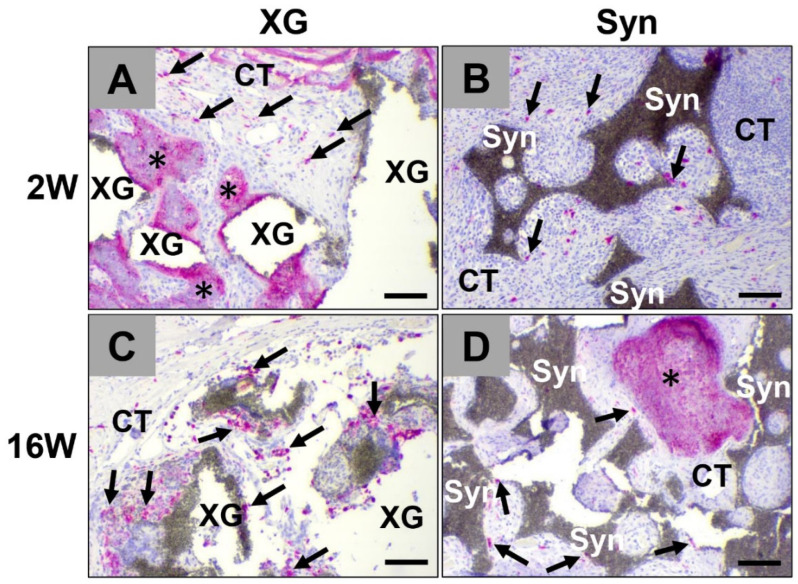
Immunohistochemical detection of anti-inflammatory macrophages within the implantation beds of both BSM at (**A**,**B**) 2 weeks and (**C**,**D**) 16 weeks post implantation. Black arrows: CD163-positive macrophages, XG: xenogeneic bone graft cerabone^®^, Syn: synthetic bone graft maxresorb^®^, black asterisks: new bone growth, CT = connective tissue (immunohistochemical CD163-staining, 10× magnification, scale bars = 100 µm).

CD163-positive anti-inflammatory macrophages were found in higher numbers within the implantation beds of the xenogeneic BSM cerabone^®^ group relative to the numbers in the synthetic BSM maxresorb^®^ (Figure 3) group. Interestingly, anti-inflammatory macrophages appeared within the granulation tissue that infiltrated the implantation beds but were not observed on the surface of the biomaterials (Figure 3).

**Figure 4:**
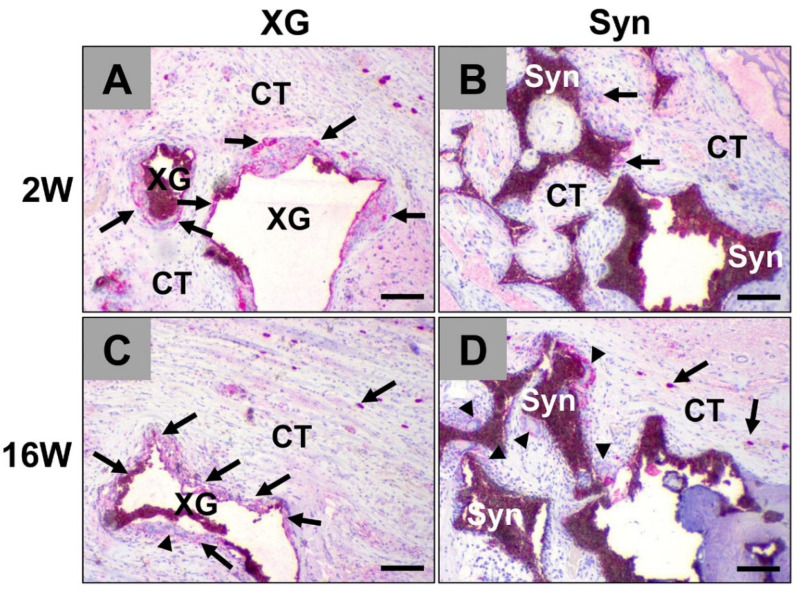
Immunohistochemical detection of pro-inflammatory macrophages within implantation beds of both BSMs including (**A**,**C**) the xenogeneic BSM cerabone^®^ (XG) and (**B**,**D**) the synthetic BSM maxresorb^®^ (Syn) at 2 weeks (**A**,**B**) and 16 weeks post implantation (**C**,**D**). Black arrows: CD11c-positive cells, black arrowheads: positive multinucleated giant cells, CT = connective tissue (immunohistochemical CD11c-detection, 10× magnification, scale bars = 100 µm).

Immunohistochemical detection of CD11c-positive pro-inflammatory macrophages revealed that positive mono- and multinucleated cells occurred mostly at the surface of the xenogeneic and synthetic BSM granules (Figure 4).

### 2.2. Scoring Results

The histopathological scoring revealed no significant differences between the values for polymorphonuclear cells, lymphocytes, plasma cells, macrophages, necrosis, neovascularization, fibrosis, or fatty infiltrate in both study groups at two weeks post implantation. At this time point, the inflammatory tissue responses were mainly composed of macrophages, followed by moderate numbers of polymorphonuclear cells and lymphocytes. A Kruskal–Wallis (K-W) test showed that, at this early time point, there were significant differences between the mean scores of multinucleated giant cells, being higher in the control material group including the xenogeneic BSM cerabone^®^ (*p* < 0.05). The scores are shown in Table 1.

The scoring evaluation revealed no significant differences between the values of polymorphonuclear cells, lymphocytes, plasma cells, macrophages, necrosis, fibrosis, or fatty infiltrate in both study groups at 16 weeks post implantation. At this time point, the inflammatory tissue responses were mainly composed of macrophages, followed by moderate numbers of polymorphonuclear cells and lymphocytes. The results of a Kruskal–Wallis (K-W) test showed that, at this late study time point, there were significant differences between the mean scores of multinucleated giant cells (*p* = 0.001), being higher for the test material group including the synthetic BSM maxresorb^®^. Furthermore, the neovascularization mean scores were significantly higher in the test material group (*p* = 0.0025). The scores are presented in Table 1.

Calculation of irritancy scores revealed that the test material maxresorb^®^ had an average treatment irritancy score of 12.21, and the control material cerabone^®^ had an average treatment irritancy score of 11.03. Thus, the overall irritancy score for the test material maxresorb^®^ was 1.18 and this material was considered non-irritant at two weeks post implantation, as shown in Table 2.

At 16 weeks post implantation, the test material maxresorb^®^ had an average treatment irritancy score of 13.71, and the control material cerabone^®^ had an average treatment irritancy score of 11.05. Thus, the overall irritancy score for the test material maxresorb^®^ was 2.66 and this material was considered non-irritant at this study time point, as shown in Table 2.

### 2.3. Histomorphometrical Analyses

#### 2.3.1. Bone Regeneration

**Figure 5:**
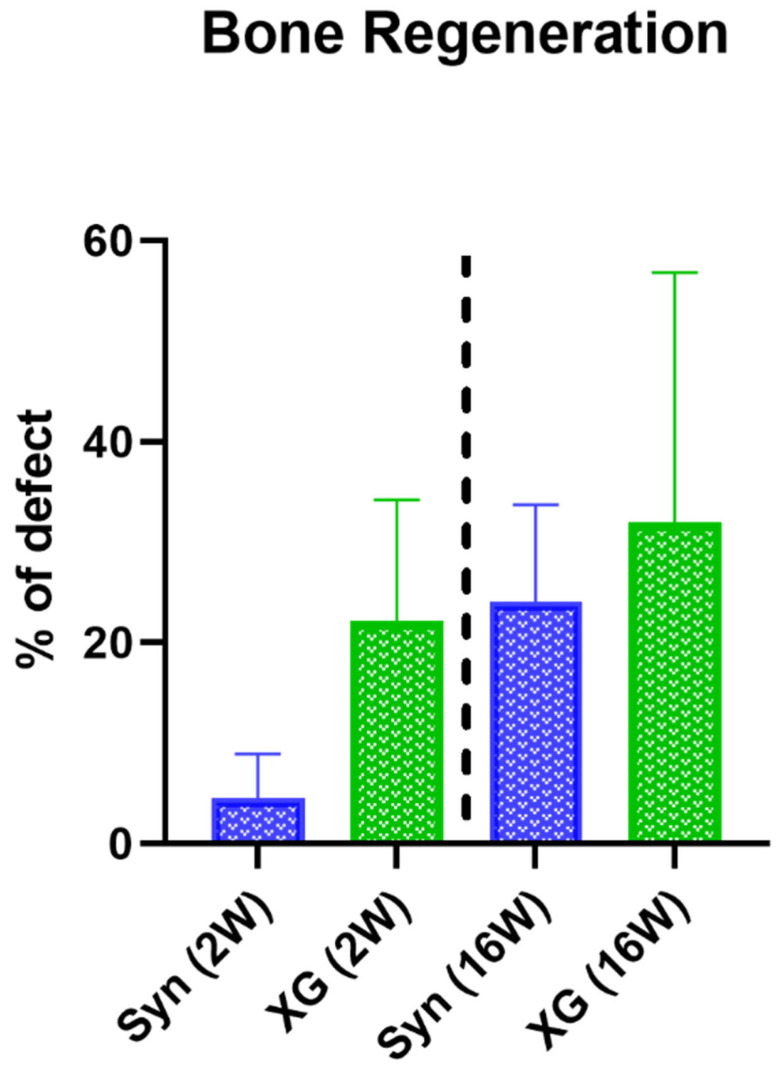
Histomorphometrical results of bone regeneration at 2 and 16 weeks post implantation (*n* = 10).

The histomorphometrical measurement of bone regeneration showed no significant differences between the test and control groups at both study timepoints (Table 3 and Figure 5).

#### 2.3.2. Immune Response

**Figure 6:**
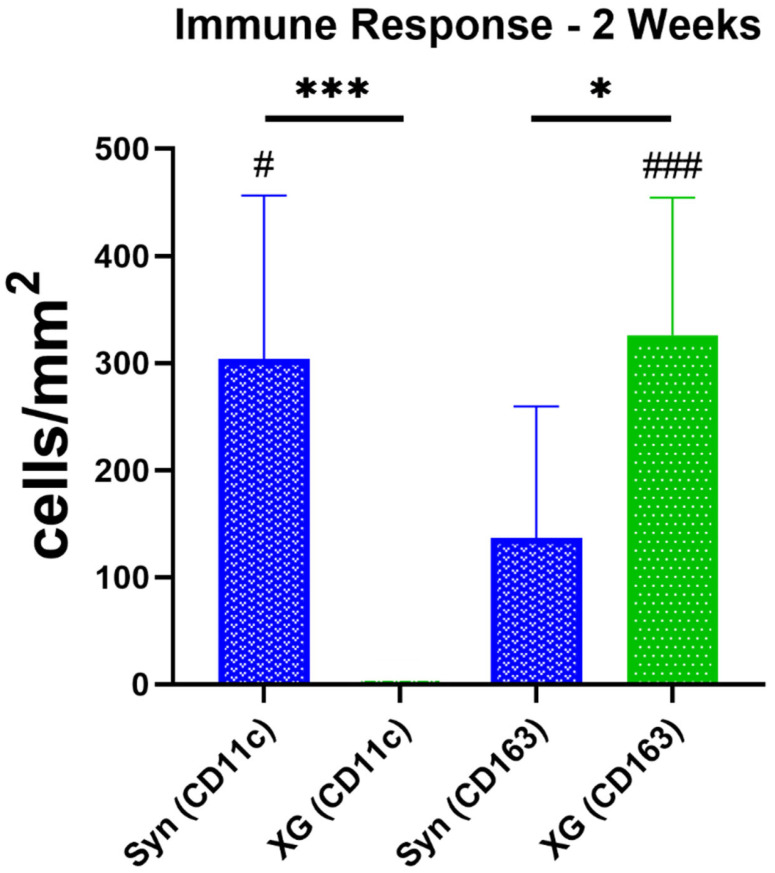
Results of the immune response analysis at 2 weeks post implantation. (Interindividual differences: * *p* < 0.05, *** *p* < 0.001, intraindividual differences: # *p* < 0.05, ### *p* < 0.001) (*n* = 10).

The histomorphometrical measurements of macrophage subtypes revealed that, at two weeks post implementation, significantly lower numbers of pro-inflammatory cells were detected in the xenogeneic BSM cerabone^®^ group than in the synthetic BSM maxresorb^®^ group (*** *p <* 0.001 (Figure 6). Additionally, significantly higher numbers of anti-inflammatory macrophages were found in the xenogeneic group compared to the pure synthetic BSM group (* *p <* 0.05). Interindividual statistical analysis showed that, in the synthetic BSM group, significantly higher numbers of pro-inflammatory macrophages (# *p* < 0.05) were measured at two weeks post implementation (Figure 6). In contrast, at this early study time point, significantly higher numbers of anti-inflammatory macrophages were found in the xenogeneic BSM group (### *p <* 0.001) (Figure 6).

**Figure 7:**
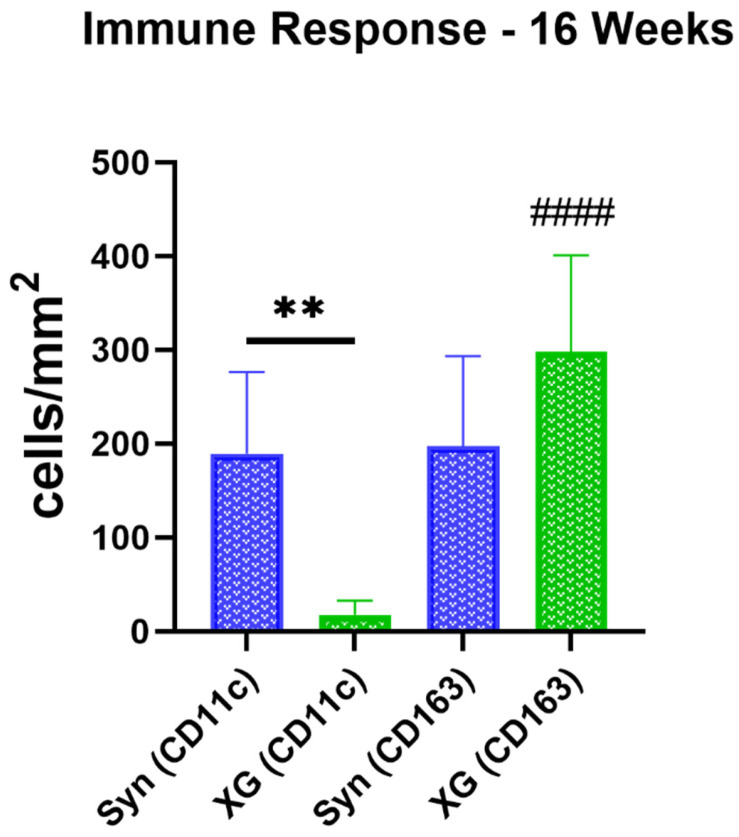
Results of the immune response analysis at 16 weeks post implantation. (Interindividual differences: ** *p* < 0.01, intraindividual differences: #### *p* < 0.0001) (*n* = 10).

Statistical analysis at 16 weeks post implantation showed significantly higher numbers of pro-inflammatory macrophages in the synthetic BSM maxresorb^®^ group compared to the xenogeneic BSM cerabone^®^ group (** p < 0.01) (Figure 7). Additionally, higher numbers of anti-inflammatory macrophages compared to the anti-inflammatory subtype were detected in the xenogeneic BSM group (*#### p <* 0.0001). All data is shown in Table 4.

## 3. Discussion

Synthetic bone substitute materials (BSM) have been proposed as alternatives to autologous, but also to allogeneic and xenogeneic, materials for a broad variety of clinical indications [5]. The indications for both BSM types are mainly dictated by their different material properties. Thus, synthetic materials undergo a degradation pattern based on their chemical composition [5,18]. Most synthetic BSMs are composed of calcium phosphates, such as hydroxyapatite (HA) and beta-tricalcium phosphate (β-TCP), both compounds having been found to be more effective than other calcium phosphate compounds, such as alpha-TCP and a variety of other synthetic substances [5,18]. Mixtures of HA and beta-TCP in different ratios in the form of so-called biphasic materials have been found to be particularly effective as the combination of the two phases leads to “mixed” or adapted degradation behavior, making such mixtures suitable for different clinical indications [5,18]. With respect to foreign body reaction to BSMs, including the production of phagocytes, such as macrophages and multinucleated giant cells (MNGCs), as correlates of the process of cellular resorption, TCP was found to attract significantly higher numbers of MNGCs compared to HA [19,20,21]. Interestingly, a biphasic BSM was observed to induce significant giant cell formation comparable to a TCP-group within the first 15 days post implantation, while the induction of phagocytes was comparable to the HA-group after 15 days [22]. In contrast, it was found that xenogeneic BSM induced phagocytes predominantly in the early phase after application followed by a decrease after some weeks, indicating that this material class does not undergo long-term phagocytic activity and provides so-called volume stability [23]. Thus, different tissue reaction patterns have been described in the literature, while both materials seem to lead to comparable regenerative results [22,23]. Therefore, the initial aim of the present study was to compare the tissue reaction pattern to both materials based on the DIN ISO 10993-6 scoring system.

It is still unclear if both material classes might also induce a different alignment of the inflammatory tissue response. In this context, macrophages and multinucleated giant cells are key elements of the cellular biodegradation of BSM and are involved in the molecular cascade towards biomaterials via the expression of a broad variety of pro- and anti-inflammatory molecules whose overall alignment seems to determine the outcome of the material-associated regeneration process [8,11,12,24,25]. In general, it is assumed that medical devices, such as the BSMs analyzed, i.e., the synthetic BSM maxresorb^®^ and the xenogeneic BSM cerabone^®^, trigger an overall anti-inflammatory cell cascade phenotype and can optimally support (bone) tissue regeneration [5,26]. Therefore, an additional objective of this study was the histomorphometrical measurement of macrophage subtypes within the implantation beds of both BSMs.

A standardized calvaria implantation model in Wistar rats, in combination with established histological, histopathological, and histomorphometrical methods, was conducted to investigate the two questions posed above [17].

Initially, histopathological analysis revealed that the synthetic and xenogeneic BSMs exhibited a comparable tissue response, as has been previously reported [7,17,22,23]. Briefly, application of the synthetic BSM maxresorb^®^ resulted in a slightly increased occurrence of phagocytes, i.e., both macrophages and multinucleated giant cells, compared to the xenogeneic bone substitute cerabone^®^. In this context, it has been found in studies that evaluated BSMs that synthetic BSMs, such as the biphasic material analyzed in the present study, exhibited a higher cellular resorption pattern as also found here [22,23]. The dissolution behavior might contribute to the sustained degradation behavior of synthetic BSMs, whereby calcium and phosphate ions are sustainably released over time [27,28].

Despite the differences described above, as well as the differences determined by scoring in accordance with DIN EN ISO 10993-6, the test material (maxresorb^®^) was assessed as non-irritant compared to the control material (cerabone^®^). Taking into account all the results of the present study, and considering the local effects after intraosseous implantation, it can be concluded that the synthetic BSM was assessed to be non-irritant according to DIN EN ISO 10993-6:2017. This result is consistent with the findings of a broad number of studies that have reported its successful clinical application [29,30,31].

These results are also consistent with histomorphometrical measurements of the bone regeneration process taken that showed comparable quantities of the newly formed matrix within the implantation beds of both materials at both study time points. Although there was a tendency towards decreased initial bone growth in the synthetic BSM group, no significant differences were found. Thus, the measurement results underline the comparable biocompatibility of both BSMs. This observation may be due not least to the chemical similarity of the two materials. The synthetic biphasic material consisted of 60% HA and 40% β-TCP, i.e., two calcium phosphate phases, while the xenogeneic material consisted of 100% “natural” HA.

The results suggest that the observed slight differences in tissue responses have no bearing on the healing capacity of the two substitute materials and confirm the validity of the scoring system for the DIN ISO standard, demonstrating the biocompatibility of the two materials by direct comparison.

Analysis of the immune response in the present study showed that the synthetic BSM maxresorb^®^ induced a significantly higher pro-inflammatory macrophage response. In contrast, a significantly higher number of anti-inflammatory macrophages was detected in the xenogeneic BSM cerabone^®^ group at two weeks post implantation. Significantly higher numbers of pro-inflammatory macrophages were still found at 16 weeks post implantation in the synthetic BSM group, while no differences in the induction of anti-inflammatory macrophage numbers were observed. This data leads to the conclusion that the xenogeneic BSM induced higher anti-inflammatory tissue responses, while the synthetic material induced higher pro-inflammation tissue responses. This observation has also been previously made by Rolvien and Barbeck et al. [32]. Their results showed that material degradation was mainly carried out by pro-inflammatory cells of the macrophage and MNGC lines and introduced the hypothesis that the degradation of bone substitutes was directly associated with the occurrence of pro-inflammatory cells. In this context, it has also been observed that the biodegradation of BSMs, such as the analyzed synthetic material, is often connected with the cellular production of reactive oxygen species (ROS), which are involved in the progression of inflammatory conditions [33]. Interestingly, the authors assumed that a local pro-inflammatory milieu during material degradation might not be compensated by an equally high anti-inflammatory tissue response so that the material-induced immune response might lead to regenerative failure. The results of the present study disprove this assumption and show that the application of degradable BSMs, such as the investigated material, also induces a higher pro-inflammatory tissue response, but this is flanked by comparably high anti-inflammatory activity. Thus, even in the case of synthetic BSMs, a balanced degree of inflammation is present, which, in addition to their good osteoconductive properties, underlines the biocompatibility of these materials. Moreover, there were no to very slight differences overall compared to the tissue reactions of the xenogeneic material, which again confirms this assumption.

Combining the methodologies of biomaterial scoring in accordance with the DIN ISO standard and histomorphometric analysis for the immunohistochemical detection of the two macrophage phenotypes expands the means by which biomaterials may be analyzed. In the case of the histopathological scoring, the presence of macrophages is viewed negatively, with increased macrophage score corresponding to an increased irritancy score. However, the alternatively activated, so-called anti-inflammatory macrophages have been widely reported to enhance tissue and wound repair [11,12,24,25,26]. It is important to recognize that classically activated, so-called pro-inflammatory macrophages occur as phagocytes in various physiological conditions and are involved in the resorption of pathogens or extracellular matrix components and other agents [12,34,35]. Thus, application of immunohistochemical detection approaches can lead to the conclusion that a pro-inflammatory environment could be a precursor to biodegradation rather than “negative” inflammatory tissue reactions that might hinder biocompatibility. Furthermore, this data leads to the conclusion that a balance in macrophage numbers with pro- and anti-inflammatory phenotypes is necessary for the successful application of a biocompatible material, which is in contradiction to the common narrative in this field that macrophages are always adverse and cause “bioincompatibility”. The tissue response to a biomaterial must be tailored to its application or “purpose”. Hence, biocompatibility should be redefined as such rather than as a description of the inflammatory state of the surrounding tissue of the biomaterial. It is an individualized characteristic that changes in definition depending on the intended application of the biomaterial.

Overall, the results of the present study confirm the biocompatibility of the synthetic BSM maxresorb^®^ and lead to the conclusion that this material class is a suitable alternative to natural BSMs, such as the analyzed xenogeneic material cerabone^®^, for a broad range of indications.

## 4. Materials and Methods

### 4.1. Biomaterials

#### 4.1.1. Xenogeneic Bone Substitute Material

Cerabone^®^ (botiss biomaterials GmbH, Zossen, Germany) is a xenogeneic BSM obtained from bovine femoral heads, which is subjected to heat treatment [3]. The procedure includes a heating step at temperatures that reach 1250 °C [3]. The granule size range of this BSM is 0.5–1.0 mm. cerabone^®^ is gamma-sterilized.

#### 4.1.2. Synthetic Bone Substitute Material

Maxresorb^®^ (botiss biomaterials GmbH, Zossen, Germany) is a biphasic synthetic BSM that is composed of two phases of calcium phosphates (60% hydroxyapatite and 40% ß-tricalcium phosphate). The manufacturing process is based on the preparation of a homogeneous, ceramic slurry. The characteristic porosity of the material is accomplished by aeration of the slurry resulting in a porous and interconnected microstructure with defined macropores (200–800 µm) and micropores (1–10 µm). The granule size of maxresorb^®^ is 0.5–1.0 mm. This BSM is gamma-sterilized.

### 4.2. In Vivo Study

For the in vivo study, 20 6–8-week-old Wistar rats were initially obtained from the Military Medical Academy (Belgrade, Serbia). Afterwards, the experimental animals were randomly allocated to two study groups to compare the tissue responses at 2 and 16 weeks post implantation. The materials were implanted according to the DIN ISO 10993-6 standard [15]. Every study group included 10 defect sites per group and timepoint.

The in vivo experiments and animal housing were conducted at the Faculty of Medicine (University of Niš, Serbia). The animals were kept under standard conditions (water ad libitum, artificial light, and regular rat pellets) and standard pre- and postoperative care were ensured. Experiments were authorized by the local ethical committee of the Faculty of Medicine (University of Niš, Serbia) based on the approval of the Veterinary Directorate of the Ministry of Agriculture, Forestry and Water Management of the Republic of Serbia (approval number 323-07-00073/2017-05/7; date of approval: 22 February 2017).

### 4.3. Implantation and Explantation Procedures

The animals underwent implantation of the BSMs for two study time periods, i.e., 2 and 16 weeks. The implantations were conducted as follows based on a standardized implantation procedure that was already published [17]: Initially, the animals were anesthetized by means of an intraperitoneal injection of ketamine [100 mg/kg of body weight] and xylazine [5 mg/kg of body weight] followed by shaving of the implantation side and disinfection. Afterwards, the surgical field was prepared by midline sagittal incisions combined with anterior and posterior subperiosteal dissections. Following this step, the frontal and parietal regions of the calvarias were exposed and bilateral cranial bone defects (2 defects/calvaria) with a diameter of 5 mm were created by means of a trephine bur (GC, Tokyo, Japan). Afterward, the biomaterials were inserted following a planned scheme. Finally, the defects were covered by a collagen membrane (Jason^®^ membrane, botiss biomaterials GmbH, Berlin, Germany) and sutured.

### 4.4. Histological Preparation and Staining Methods

The calvarial explants were cut into segments containing the left and right defects, including the biomaterials. To start the histological processing, the explants were inserted in an automated tissue processing device (SLEE medical GmbH, Nieder-Olm, Germany) that dehydrated the samples, preparing them for plastic embedding. After dehydration, stepwise immersion at 4 °C with Technovit 9100 medium using different infiltration solutions (pre-infiltration, infiltration I + II with the same composition) was conducted. Afterwards, the polymerization solution was prepared according to the operating instructions. The explants were orientated and placed on the bottom of rolled rim bottles (rolled rim bottles with snap-on lid (VWR, Darmstadt, Germany) in a fashion whereby the cutting surface was facing the bottom of the bottles, subsequently followed by pouring with the polymerization mixture. To avoid the exposition of oxygen and, therefore, the occurrence of irregular polymerization, the bottles were sealed airtight and immediately stored at −20 °C until the liquid Technovit 9100 was completely polymerized and hardened. Subsequently, the tissue blocks were trimmed into shape by means of a grinding machine (EcoMet 30, Buehler, Esslingen, Germany). Sections with a thickness of 4–6 µm were prepared using a rotation microtome (CUT4060E, microTec GmbH, Walldorf, Germany). Histochemical and immunohistochemical stainings were made using specialized methods, as previously published [16,17]. A single section of every explant was used for hematoxylin and eosin staining. Two additional sections were used for immunohistochemical staining of CD11c and CD163 cell markers. Briefly, antibodies for detection of pro- and anti-inflammatory macrophage subtypes, i.e., integrin alpha x (CD11c) (abx231412, Abbexa Ltd., Milton, UK) and hemoglobin scavenger receptor (CD163) (ab182422, abcam, Cambridge, UK), were used to assess the immunological tissue response. CD11c is a complement receptor which is often exploited as a marker molecule to track monocytes and macrophages. This marker molecule has been shown to allow separation between pro-inflammatory macrophages and other cells involved in the foreign body reaction to biomaterials [17,36,37]. Furthermore, it has been demonstrated that CD163 is a marker of anti-inflammatory macrophages that can be used to distinguish both macrophage subtypes [7,38,39].

Initially, the slides were treated with TRIS-EDTA pH 9 for 20 min in a steamer at 96 °C, followed by equilibration using a cold wash buffer. Before incubation with the respective first antibody for 60 min at room temperature, a blocking step with protein blocking solution for 10 min was conducted. Final detection of the antigen was enabled by incubation with the biotinylated secondary antibody for 15 min, followed by application of the streptavidin–alkaline–phosphatase conjugate and the permanent alkaline phosphatase (AP)-red chromogen. Finally, counterstaining was performed using Mayer’s hemalum solution (Merck KGaA, Darmstadt, Germany). Unless otherwise stated, all solutions and reagents were purchased from Zytomed Systems (Berlin, Germany).

### 4.5. Histopathological and Histomorphometrical Analyses

The implantation beds of both BSMs were assessed qualitatively for immune response, state of biomaterial, and bone regrowth. The histopathological evaluation of the tissue reactions to the implanted biomaterials was conducted based on a previously described protocol according to the DIN EN ISO 10993-6:2017 guidelines [15]. The synthetic BSM was declared as a test article, while the xenogeneic material was used as a control device. The slides were evaluated to assess the local tissue healing response. Sections of the implant sites were evaluated for a number of parameters evaluating the safety of the test and control material groups. The HE-stained sections were analyzed and graded according to cell type and responses. Biocompatibility was evaluated following the irritancy/reactivity grading scheme included in the DIN EN ISO 10993-6: 2016 Annex E, included in Table 5 below. The tissue reactivity associated with the respective biomaterial was evaluated in accordance with the ISO scoring system to obtain a scoring value of the reactivity grade within the peri-implant tissue.

The irritancy/reactivity scores (see Table 6) based on DIN EN ISO 10993-6:2017 derived from the parameters listed in Table 1 were calculated as follows for each defect:

Irritancy score (for each implantation site) = (Polymorphonuclear Cells + Lymphocytes + Plasma Cells + Macrophages + Giant Cells + Necrosis) × 2 + (Neovascularization + Fibrosis + Fatty Infiltrate).(1)

The irritancy scores for each test or control treatment were then calculated by averaging the irritancy scores of all test or control implantation sites for each treatment. Each irritancy/reactivity score was calculated as follows:

Test Article irritancy score − Control Articles irritancy score = irritancy/reactivity score.(2)

If the result was a negative number, the irritancy/reactivity score was considered to be 0.0.

**Figure 8:**
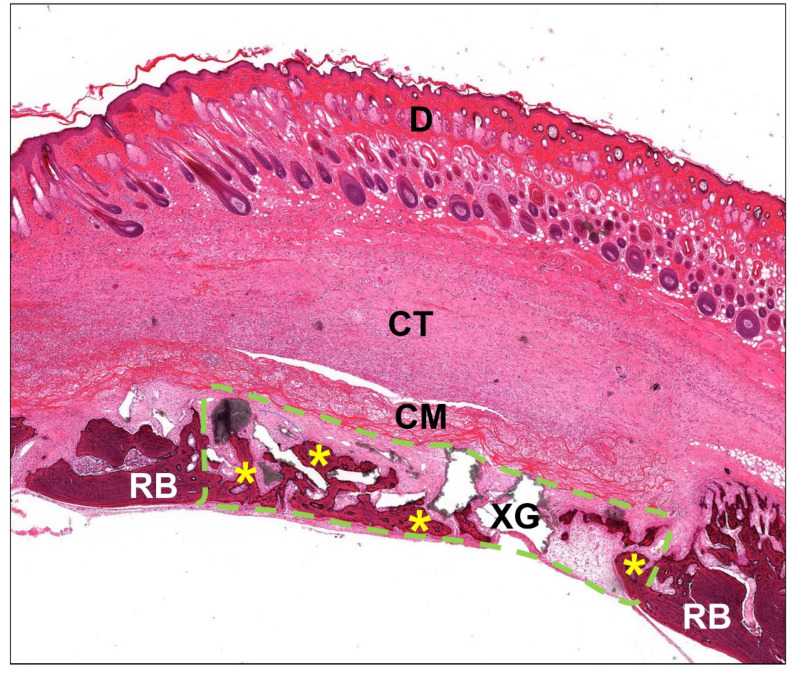
Exemplary overview of the bony implantation bed of the xenogeneic BSM (XG) at 2 weeks post implantation. The defect area including the implanted BSM (green dashed line) was used as the basis for the histomorphometrical measurements. Yellow asterisks = newly formed bone, RB = residual bone, CM = collagen membrane, CT = connective tissue, D = dermis (HE-staining, “total scan”, 100× magnification).

Additionally, the histomorphometrical measurements of the occurrence of macrophage subtypes and bone growth were conducted via specialized digital methods described by Lindner et al. [16]. Briefly, all respective slides were scanned using a digital scanning microscope (M8, Precipoint, Munich, Germany). The regions of interests, i.e., the defect area including the implanted BSM, soft tissue, new bone growth, etc., were manually marked and measured using ImageJ (National Institutes of Health, Bethesda, MD, USA) (Figure 8).

Immunohistochemically stained cells were counted using an especially developed ImageJ plugin, which has been described previously [16]. For this measurement step, the workflow-plugin enabled calculation of the respective cell densities in relation to the total implant area (cells/mm^2^).

### 4.6. Statistical Analysis

For analysis of the scoring data, normality was tested using the Shapiro–Wilk normality test followed by a statistical analysis using the Kruskal–Wallis test to compare the grades between the two groups. Statistical significance was defined as * *p* < 0.05.

For analysis of the histomorphometrical data, analysis of variance (ANOVA) was performed using GraphPad Prism 8.0 software (GraphPad Software Inc., La Jolla, CA, USA), followed by an LSD post hoc test for statistical analysis of the qualitative data obtained via histomorphometry. Both inter- (*) and intraindividual (#) significances were calculated and designated as significant if the *p*-values were less than 0.05 (*/# p < 0.05), and highly significant if the *p*-values were less than 0.01 (**/## p < 0.01), less than 0.001 (***/### p < 0.001), or less than 0.0001 (****/#### p < 0.0001). Finally, the data were graphed in the form of means and standard deviations.

## 5. Conclusions

The present study was conducted to analyze in vivo responses to both xenogeneic and synthetic bone substitute granules. The results of the present study showed that the application of the synthetic BSM maxresorb^®^ induced a higher pro-inflammatory tissue response, while the xenogeneic BSM cerabone^®^ induced a higher anti-inflammatory reaction. Additionally, comparable bone regeneration amounts were found in both study groups. However, the histopathological scoring revealed that the synthetic BSM exhibited non-irritant scores at all timepoints compared to the tissue responses of the xenogeneic BSM that was used as the control. Overall, the results of the present study indicate the biocompatibility of synthetic BSMs and lead to the conclusion that this material class is a suitable alternative to natural BSMs, such as the analyzed xenogeneic material, for a broad range of indications.

## Figures and Tables

**Table 1:** Results of the scoring evaluation (of test and control materials) at 2 and 16 weeks post implantation (*n* = number of sites).

Parameter	Mean ± SD-Inflammation and Inflammatory Cell Types			
	2 Weeks		16 Weeks	
	Maxresorb^®^	Cerabone^®^	Maxresorb^®^	Cerabone^®^
	(*n* = 10)	(*n* = 10)	(*n* = 10)	(*n* = 10)
Polymorphonuclear Cells	1.2 ± 0.3	1.2 ± 0.5	0.9 ± 0.3	0.9 ± 0.1
Lymphocytes	1.3 ± 0.3	1.3 ± 0.2	1.8 ± 0.5	2.2 ± 0.3
Plasma Cells	0.0 ± 0.0	0.0 ± 0.0	0.0 ± 0.0	0.0 ± 0.0
Macrophages	3.0 ± 0.0	3.0 ± 0.0	2.9 ± 0.1	3.0 ± 0.0
Giant Cells	0.6 ± 0.2	1.3 ± 0.4	0.5 ± 0.2	1.1 ± 0.2
Neovascularization	1.1 ± 0.2	1.1 ± 0.2	1.3 ± 0.3	1.0 ± 0.1
Fibrosis	0.0 ± 0.0	0.0 ± 0.1	0.0 ± 0.0	0.0 ± 0.0
Fatty infiltrate	0.0 ± 0.0	0.0 ± 0.0	0.0 ± 0.0	0.0 ± 0.0
Necrosis	0.1 ± 0.1	0.1 ± 0.3	0.0 ± 0.0	0.0 ± 0.0
Polymorphonuclear Cells; Lymphocytes; Plasma Cells; Macrophages; Giant Cells Scoring Matrix: 0 = 0; 1 = Rare, 1–5 per high powered (400×) field (phf) (giant cells = 1–2/phf); 2 = 6–10/phf (giant cells = 3–5/phf); 3 = Heavy infiltrate; 4 = Packed.				

**Table 2:** Irritancy scores and irritancy status at 2 and 16 weeks post implantation. Test device: maxresorb^®^, control device: cerabone^®^.

	Irritancy Scores	
	2 Weeks (*n* = 10)	16 Weeks (*n* = 10)
Treatment Irritancy Score of Test Device-maxresorb^®^	11.03	13.71
Treatment Irritancy Score of Control Device-cerabone^®^	12.21	11.05
Overall Irritancy Score of Test Device-maxresorb^®^	1.18	2.66
Irritancy	Non-irritant	Non-irritant

**Table 3:** Amounts of newly formed bone tissue in the two study groups and at the two study timepoints (as percentages of defect area) (*n* = 10).

Material Group/Timepoint	2 Weeks	16 Weeks
cerabone^®^ (XG)	22.18 ± 12.05	31.98 ± 24.83
maxresorb^®^ (Syn)	4.53 ± 4.41	24.10 ± 9.61

**Table 4:** Mean values of CD163-positive anti-inflammatory and CD11c-positive pro-inflammatory macrophage subtypes per mm^2^ within the implantation beds of all material groups (*n* = 10).

	Cerabone^®^ (XG)		Maxresorb^®^ (Syn)	
	CD163+	CD11c+	CD163+	CD11c+
2 weeks	326.2 ± 128.1	9.503 ± 14.14	137.0 ± 122.3	304.0 ± 152.4
16 weeks	298.2 ± 103.0	17.59 ± 15.09	197.4 ± 96.03	189.5 ± 86.97

**Table 5:** Histologic Evaluation System for Irritancy/Reactivity Cell Type/Response. Adapted from DIN EN ISO 10993-6 [15]. phf = per High Powered (×400) Field.

Response	Score (Phf = Per High Powered (400×) Field)				
	0	1	2	3	4
Polymorphonuclear cells	0	Rare, 1–5/phf	6–10/phf	Heavy infiltrate	Packed
Lymphocytes	0	Rare, 1–5/phf	6–10/phf	Heavy infiltrate	Packed
Plasma cells	0	Rare, 1–5/phf	6–10/phf	Heavy infiltrate	Packed
Macrophages	0	Rare, 1–5/phf	6–10/phf	Heavy infiltrate	Packed
Giant cells	0	Rare, 1–2/phf	3–5/phf	Heavy infiltrate	Packed
Necrosis/osteolysis	0	Minimal	Mild	Moderate	Marked
Neovascularization	0	Minimal capillary proliferation focal, 1–3 buds	Groups of 4–7 capillaries with supporting fibroblastic structures	A broad band of capillaries with supporting structures	Extensive band of capillaries with supporting fibroblastic structures
Fibrocytes/fibroconnective tissue, fibrosis	0	Narrowband	Moderately thick band	Thick band	Extensive band
Fatty infiltrate	0	A minimal amount of fat associated with fibrosis	Several layers of fat and fibrosis	Elongated and broad accumulation of fat cells about the implant site	Extensive fat completely surrounding the implant
Irritancy score = (Polymorphonuclear Cells + Lymphocytes + Plasma Cells + Macrophages + Giant Cells + Necrosis) × 2 + (Neovascularization + Fibrosis + Fatty Infiltrate).					

**Table 6:** Irritancy/Reactivity Grade. Adapted from the DIN EN ISO 10993-6 [15].

Overall Irritancy Score	Irritancy/Reactivity Status
0.0 to 2.9	Minimal or no reaction (non-irritant)
3.0 to 8.9	Slight reaction (slight irritant)
9.0 to 15.0	Moderate reaction (moderate irritant)
>15.1	Severe reaction (severe irritant)

## Data Availability

All data are included in the manuscript.
